# Synergistic Effect of Combination of a Temoporfin-Based Photodynamic Therapy with Potassium Iodide or Antibacterial Agents on Oral Disease Pathogens In Vitro

**DOI:** 10.3390/ph15040488

**Published:** 2022-04-18

**Authors:** Yin-Hwa Shih, Cheng-Chia Yu, Kai-Chi Chang, Yu-Hsin Tseng, Po-Jung Li, Shih-Min Hsia, Kuo-Chou Chiu, Tzong-Ming Shieh

**Affiliations:** 1 Department of Healthcare Administration, Asia University, Taichung 41354, Taiwan; evashih@gm.asia.edu.tw; 2 School of Dentistry, Chung Shan Medical University, Taichung 40201, Taiwan; ccyu@csmu.edu.tw; 3 Department of Dentistry, Chung Shan Medical University Hospital, Taichung 40201, Taiwan; 4 Institute of Oral Sciences, Chung Shan Medical University, Taichung 40201, Taiwan; 5 School of Dentistry, China Medical University, Taichung 40402, Taiwan; ketty60221@gmail.com (K.-C.C.); ll820731@gmail.com (P.-J.L.); 6 Department of Pediatrics, Kaohsiung Medical University Hospital, Kaohsiung 80756, Taiwan; grapepuff@gmail.com; 7 School of Nutrition and Health Sciences, Taipei Medical University, Taipei 110301, Taiwan; bryanhsia@tmu.edu.tw; 8 Division of Oral Diagnosis and Family Dentistry, Tri-Service General Hospital, National Defense Medical Center, Taipei 11490, Taiwan

**Keywords:** biofilm, hypoxia, MRSA, photodynamic therapy (PDT), temoporfin

## Abstract

5, 10, 15, 20-Tetrakis(3-hydroxyphenyl)chlorin (temoporfin) is a photosensitizer used in photodynamic therapy for oral cancer and periodontal disease treatment. This study determined the minimum inhibitory concentrations (MICs) and minimum bactericidal concentrations (MBCs) of temoporfin. Additionally, the combination of potassium iodide (KI) or antimicrobial agents in oral pathogens under hypoxic or normoxic conditions were determined. We also evaluated the biofilm removal effect and detected the expressions of the antibiotic resistance-related genes and biofilm formation-related genes of methicillin-resistant *staphylococcus aureus* (MRSA). The results provided reveal that the combination of the temoporfin and KI had a synergistic effect of reducing the MICs and MBCs of *Lactobacillus acidophilus* and *Lactobacillus paracasei* under normoxic and hypoxic conditions due to increasing H_2_O_2_ production. Temoporfin increased the biofilm removal of *Aggregatibacter actinomycetemcomitans*, *Enterococcus faecalis*, and *Staphylococcus aureus* under normoxic condition, and it reduced the antibiotic resistance-related genes expression of MRSA. The combination of temoporfin with ampicillin or chlorhexidine significantly enhanced the bactericidal effect on MRSA. This study provides a potential application of temoporfin on the clinical side against oral pathogens and the prevention of oral diseases.

## 1. Introduction

The oral cavity provides an optimal environment for the growth and survival of various microbes, which exist primarily as biofilm [1,2,3]. Oral pathogens form a biofilm on the surfaces of teeth, commonly known as dental plaque. Dental plaque is colonized by complex, relatively specific, and strongly interdependent microorganisms. They include aerobic and anaerobic Gram-positive bacteria, Gram-negative bacteria, and fungus. Some microbes have been implicated in oral diseases, such as caries, periodontitis, and oral candidiasis [3,4,5]. Therefore, antibiotics and antiseptic agents, such as ampicillin and chlorhexidine (CHX), are often used to treat oral diseases. However, there are side effects, and resistance to antibiotics and antiseptic agents develops upon long-term treatment. The World Health Organization (WHO) published a list of priority antibiotic-resistant pathogens in 2017, including *A. baumannii*, *P. aeruginosa*, *Enterobacteriaceae*, *E. faecium*, MRSA, *Helicobacter pylori*, *Campylobacter* spp., *Salmonella* spp., *Neisseria gonorrhoeae*, *Streptococcus pneumoniae*, *Haemophilus influenzae*, and *Shigella* spp. Except for *E. faecium*, *S. aureus*, and *Streptococcus pneumoniae*, most of the pathogens are Gram-negative. Gram-negative bacteria have thick impermeable outer membranes and are more resistant to small molecules’ diffusion than Gram-positive bacteria [6]. Antibiotic-resistant bacteria are hazardous to human health. Therefore, research is dedicated to finding alternative strategies such as synergistic compounds or new drugs against antibiotic-resistant bacteria [7,8].

The efficacy of photodynamic therapy (PDT) depends on the nontoxic photosensitizer (PS), the specific wavelength of light, and oxygen content in the organism. After activating the photosensitizer at a specific wavelength, the excitation of the PS forms an excited triplet state, which transfers energy to the surrounding molecules, generally to molecular oxygen, to form reactive oxygen species (ROS), singlet oxygen, and hydroxyl radicals. These unstable substances can cause damage to biomolecules and cause oxidation of cellular structures, leading to the death of cells, bacteria, and drug-resistant pathogens [9,10,11,12]. Therefore, PDT has been introduced into the dental field as an important new treatment for superficial precancerous oral lesions, oropharyngeal carcinoma, periodontal disease, and root canal infection [13,14,15]. Antibacterial photodynamic therapy (aPDT) is another choice for suppressing bacterial growth in the clinic.

5, 10, 15, 20-Tetrakis(3-hydroxyphenyl)chlorin (temoporfin) is presently one of the most effective hydrophobic second-generation photosensitizers for clinical PDT [16,17]. In 2001, temoporfin was approved for the treatment of head and neck squamous cell carcinoma in the European Union [18,19]. Temoporfins have good photophysical properties and high singlet oxygen yield [20]. Compared with other clinically approved photosensitizers, such as hematoporphyrin derivatives and photofirin (PF), temoporfin is applied at very low drug doses (0.1 mg/kg) and energy intensity (as low as 10 J/cm^2^) for clinical PDT response [17,19,21]. At present, the application of clinical temoporfin in periodontitis and pulp infection is less studied. After laser-illumination, polymeric bone grafting material containing 20wt% temoporfin suppressed *Porphyromonas gingivalis* and *E. faecalis* [21]. An anaerobic environment enhances the bactericidal effect of temoporfin in periodontitis and pulp infection; ultrasonic activation facilitated better diffusion of temoporfin into dentinal tubules and reduced the biofilm formation in premolars [22]. Improving the antibacterial effect of temoporfin in an anaerobic environment could enhance the potential of temoporfin for oral clinical application.

Potassium iodide (KI) is a biocompatible compound that is readily available, safe, and effective as an inorganic salt [23,24,25]. Dental studies used temoporfin combined with KI to enhance the bactericidal effect of PDT. The ROS, singlet oxygen, and hydroxyl radicals produced by PDT react with the iodide anion and produce an unstable iodide radical, triiodide anion, and hydrogen peroxide to harm the cells [8,26,27,28,29]. Photosensitive reactions such as burning sense, skin redness, pain, and prolonged sun sensitivity in the treated area are common side effects of PDT. These symptoms are relieved by reducing the PDT dosage. To reduce the dosage of temoporfin treatment and find its synergistic compounds in a dental application, the present study investigated the synergistic bactericidal effects of temoporfin combined with KI or antibacterial agents.

## 2. Results

### 2.1. The Antimicrobial Activity of Temoporfin, KI, and the Combination of Temoporfin and KI

#### 2.1.1. Determination of Temoporfin Treatment Conditions

**Figure 1:**
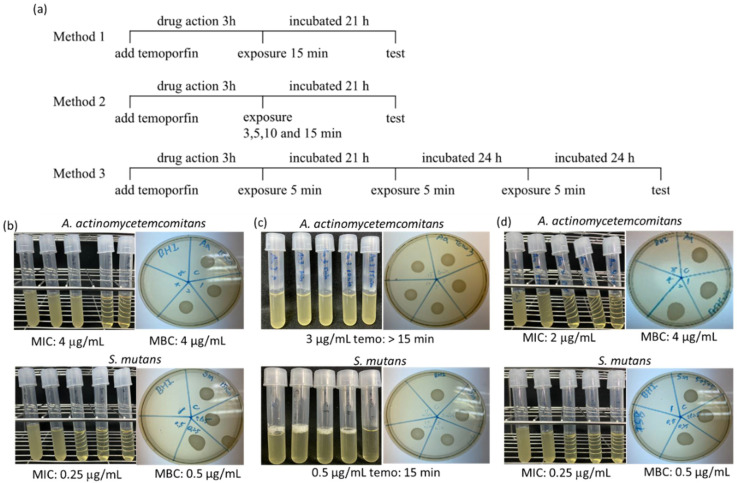
The test methods’ flow charts and outcomes of the temoporfin treatment. (**a**) The procedures for three PDT treatment methods. Values of MIC and MBC of temoporfin for *A. actinomycetemcomitans* and *S. mutans* were obtained by treatment method I (**b**), method II (**c**), and method III (**d**). The left tube figure represents MIC test; the right dish figure represents MBC test.

Three treatment conditions for temoporfin were tested using the broth dilution method on *A. actinomycetemcomitans* and *S. mutans* (Figure 1a). In method I, vehicle 1, 2, 4, and 8 μg/mL temoporfin, and vehicle 0.125, 0.25, 0.5, and 1 μg/mL temoporfin were used for *A. actinomycetemcomitans* and *S. mutans*, respectively. The exposure time was 15 min. The temoporfin’s minimum inhibitory concentrations (MICs) against *A. actinomycetemcomitans* and *S. mutans* were 4 μg/mL and 0.25 μg/mL, respectively. The minimum bactericidal concentrations (MBCs) of the temoporfin against *A. actinomycetemcomitans* and *S. mutans* were 4 and 0.5 μg/mL, respectively (Figure 1b). In method II, 3 μg/mL and 0.5 μg/mL temoporfin were used for *A. actinomycetemcomitans* and *S. mutans*, respectively, and the vehicle treatment was the control. The exposure times were 0 min, 3 min, 5 min, and 15 min. The MIC and MBC values for *A. actinomycetemcomitans* were >3 μg/mL, and for *S. mutans*, it was 0.5 μg/mL after 15 min exposure (Figure 1c). In method III, the dose of temoporfin was similar to that of the method I, and the exposure time was 5 min a day for three consecutive days. The MIC values for *A. actinomycetemcomitans* and *S. mutans* were 2 μg/mL and 0.25 μg/mL, respectively. The MBC values for *A. actinomycetemcomitans* and *S. mutans* were 4 μg/mL and 0.5 μg/mL (Figure 1d), respectively. The MIC values for *A. actinomycetemcomitans* were significantly reduced after the method III treatment compared with the method I treatment. However, there was no reduction in the MIC values for *S. mutans*. Based on the results, method I was used for further experiments in this study.

#### 2.1.2. The Kinetic Growth Curves of Temoporfin and KI Treatment in *A. actinomycetemcomitans* and *S. mutans*

KI and hydrogen peroxide reactions produce oxygen. The chemical reaction is represented as follows.

H_2_O_2_ + I^−^ → H_2_O + IO^−^

H_2_O_2_ + IO^−^ → H_2_O + O_2_ + I^−^

net equation: 2H_2_O_2_ → 2H_2_O + O_2_

**Figure 2:**
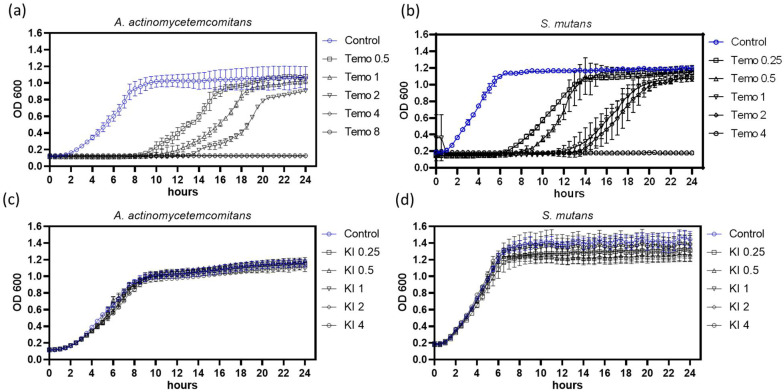
The kinetic growth curve of *A. actinomycetemcomitans* and *S. mutans* were inhibited by temoporfin in a dose-dependent manner instead of KI. *A. actinomycetemcomitans* were treated with temoporfin (**a**) and KI (**c**); similarly, *S. mutans* were treated with temoporfin (**b**) and KI (**d**). The symbols on the right side of the graphs indicate various temoporfin (µg/mL) and KI (mg/mL) doses. The blue line indicates vehicle treatment (control) in each graph.

However, it remains unclear whether this effect enhances bacterial growth or improves temoporfin’s bactericidal ability. The kinetic microplate method was used to analyze bacterial growth inhibition over 24 h, as shown in Figure 2. The clear bacterial suspensions were spread on an agar plate to double-check. No colony was defined as being completely inhibited. Kinetic analysis showed that *A. actinomycetemcomitans* and *S. mutans* growth were completely inhibited after temoporfin treatment at 4 µg/mL (Figure 2a) and 2 µg/mL (Figure 2b), respectively. A log phase delay or stationary phase delay in the growth curve after 24 h incubation implies that temoporfin inhibited bacterial growth and killed the bacteria. When treated with 0.25–4 mg/mL of KI, the growth curves of *A. actinomycetemcomitans* (Figure 2c) and *S. mutans* (Figure 2d) showed no obvious effect compared with control.

#### 2.1.3. MIC/MBC of the Temoporfin and KI Co-Treatment for Oral Bacteria under Normoxic and Hypoxic Conditions

Different bacteria have different drug absorption abilities, tolerances, and intracellular oxygen content, which may affect the efficacy of aPDT. The effect of aPDT may be affected under hypoxic conditions. In addition, we considered whether the synergistic activity of temoporfin and KI co-treatment could improve the killing effect on oral bacteria over temoporfin alone treatment. The MIC and MBC values of temoporfin and KI for the seven oral microbes are shown in Table 1. In the normoxic environment, the MIC and MBC values for *L. acidophilus*, *L. paracasei*, MRSA, and *S. mutans* were reduced by adding 1 mg/mL KI. Similarly, in the hypoxic environment, the MIC and MBC values for *A. actinomycetemcomitans*, *L. acidophilus*, *L. paracasei*, *S. aureus*, and MRSA were reduced by adding 1 mg/mL KI. In both normoxic and hypoxic environments, KI addition significantly enhanced the aPDT effect of temoporfin. KI and temoporfin co-treatment affected more bacterial species under hypoxic conditions than normoxic conditions. The co-treatment prominently reduced the MIC and MBC of *L. acidophilus* and *L. paracasei* under both normoxic and hypoxic conditions.

### 2.2. Synergistic Effect of Temoporfin Combined with Potassium Iodide (KI)

**Figure 3:**
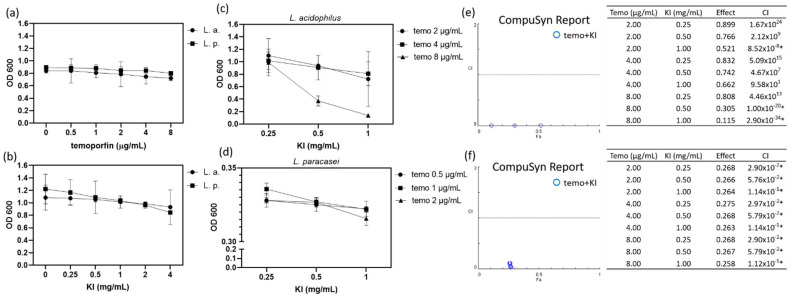
Synergistic effect of temoporfin combined with KI on *L. acidophilus and L. paracasei.* The OD600 values of bacteria after vehicle and 0.5−8 μg/mL temoporfin treatment for 24 h (**a**) and 0.25−4 mg/mL KI treatment for 24 h (**b**). The OD600 of *L. acidophilus* (**c**), and *L. paracasei* (**d**) after temoporfin and KI co-treatment for 24 h. CompuSyn report for *L. acidophilus* (**e**) and *L. paracasei* (**f**). * CI *<* 0.3.

The temoporfin and KI co-treatment showed the most significant antibacterial activity against *L. acidophilus* and *L. paracasei*. Therefore, it is important to verify these results rigorously. The dosage of 0.5–8 μg/mL temoporfin did not inhibit *L. acidophilus* and *L. paracasei* growth (Figure 3a), but the dosage of 0.25–4 mg/mL KI showed dose-dependent inhibition (Figure 3b) under hypoxic conditions. The turbidity or optical density at a wavelength of 600 nm (OD600, mean ± S. E.) of *L. acidophilus* and *L. paracasei* decreased from 1.085 ± 0.1154 to 0.9322 ± 0.1607 and from 1.22 ± 0.1378 to 0.8463 ± 0.01027, respectively. Different doses of temoporfin (2, 4, and 8 μg/mL) and KI (0.25, 0.5, and 1 mg/mL) were used for 24 h under a hypoxic environment for further analysis. The OD600 of *L. acidophilus* treated with 8 μg/mL temoporfin combined with 0.5–1 mg/mL KI was significantly decreased (Figure 3c). Similarly, the OD600 of *L. paracasei* treated with 0.5, 1, and 2 μg/mL temoporfin combined with 0.25, 0.5, and 1 mg/mL KI was significantly decreased (Figure 3d). Thus, treatment with temoporfin combined with KI, even at low doses, significantly reduced the OD600 values of *L. acidophilus* and *L. paracasei*. CompuSyn software was used to analyze whether the effect of drug combinations was synergistic or antagonistic. A combination index (CI) < 0.3 is defined as strong synergism in the CompuSyn software. The combinations of 2 μg/mL temoporfin and 1 mg/mL KI, 8 μg/mL temoporfin and 0.5 mg/mL KI, and 8 μg/mL temoporfin and 1 mg/mL KI in *L. acidophilus* (Figure 3e) showed a strong synergistic antibacterial effect. In addition, combinations of 0.5–2 μg/mL temoporfin and 0.25–1 mg/mL KI in *L. paracasei* (Figure 3f) showed a strong synergistic antibacterial effect. Temoporfin and KI co-treatment showed a synergistic antibacterial effect on *L. acidophilus* and *L. paracasei* but not on MRSA (data not shown).

### 2.3. Combination of Temoporfin with Either Ampicillin or CHX Inhibited MRSA Growth

**Figure 4:**
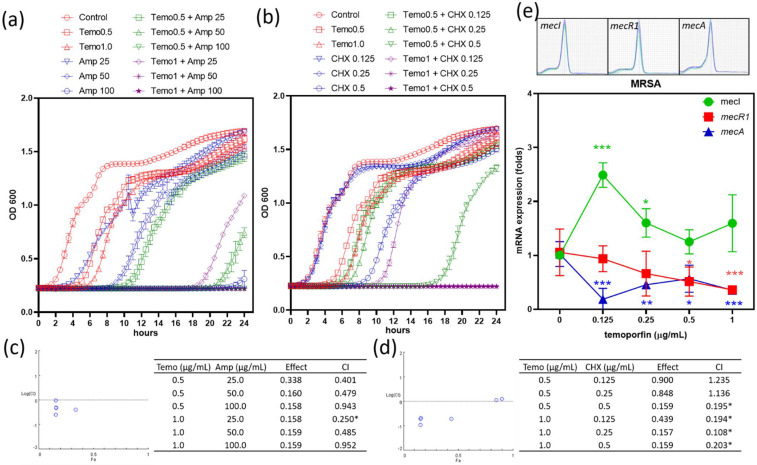
The combination of temoporfin with ampicillin or CHX inhibited MRSA growth. (**a**) The MRSA was treated with vehicle, temoporfin alone, ampicillin alone, and temoporfin and ampicillin co−treatment. (**b**) The MRSA was treated with vehicle, temoporfin alone, CHX alone, and temoporfin and CHX co−treatment. CompuSyn report for ampicillin (**c**) and CHX (**d**). (**e**) The antibiotic-resistant genes *mecI*, *mecR1*, and *mecA* expression fold change in MRSA after temoporfin treatment for 24 h. * *p <* 0.05, ** *p <* 0.01, and *** *p <* 0.001 compare with vehicle (**e**). * CI *<* 0.05 (**c**,**d**).

Although temoporfin and KI co-treatment showed no synergistic effect in MRSA, temoporfin and ampicillin co-treatment (Figure 4a) and temoporfin and CHX co-treatment (Figure 4b) clearly restricted MRSA growth. The 0.5–1 μg/mL temoporfin and 25–50 μg/mL ampicillin co-treatment slightly reduced MRSA growth, whereas the co-treatment with CHX could not reduce the growth when concentrations of CHX were 0.125–0.25 μg/mL. The 0.5–1 μg/mL temoporfin and 25–50 μg/mL ampicillin co-treatment, 0.5 μg/mL temoporfin and 0.5 μg/mL CHX co-treatment, and 1 μg/mL temoporfin and 0.125–0.25 μg/mL CHX co-treatment delayed or completely inhibited MRSA growth. The stationary phase of bacterial growth (12 h) was used to analyze the synergistic effect. All the combinations of 0.5–1 μg/mL temoporfin and 25–100 μg/mL ampicillin (Figure 4c), 0.5 μg/mL temoporfin and 0.5 μg/mL CHX, and 1 μg/mL temoporfin and 0.125–0.5 μg/mL CHX in MRSA (Figure 4d) showed synergistic antibacterial effects. Both ampicillin and CHX combined with temoporfin treatment showed a synergistic antibacterial effect on MRSA. We tested the expression of drug resistance genes in temoporfin-treated MRSA (Figure 4e). Temoporfin significantly upregulated *mecI* but downregulated *mecR1* expression. It then suppressed the expression of the antibiotic resistance gene *mecA* at 0.125–1 μg/mL temoporfin. Even at low concentrations, treatment with temoporfin considerably reduced MRSA drug resistance.

### 2.4. KI Enhanced Temoporfin Biofilm Removal Effect under Hypoxic Conditions

#### 2.4.1. The Biofilm Removal Effect of Temoporfin in a Normoxic Environment

**Figure 5:**
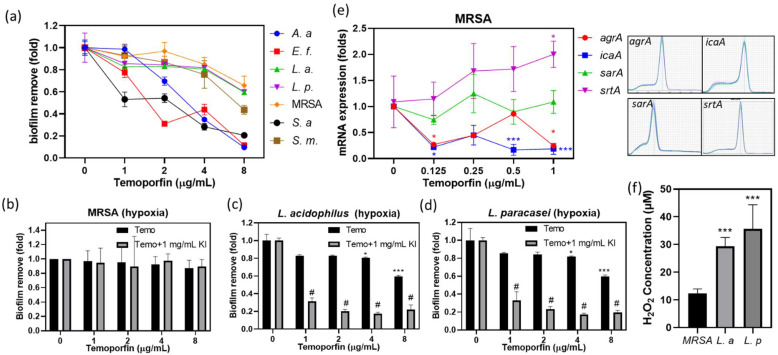
The biofilm removal effect of temoporfin and KI. (**a**) The biofilm removal ability of temoporfin in oral disease pathogens under normoxic conditions. The biofilm removal efficacy of temoporfin alone and temoporfin and KI combination against (**b**) MRSA, (**c**) *L. acidophilus*, and (**d**) *L. paracasei* biofilms under hypoxic conditions. (**e**) The biofilm formation-related gene mRNA expression after MRSA was treated with vehicle and 0.125–1 μg/mL temoporfin under hypoxic conditions. (**f**) Hydrogen peroxide production of bacteria under anaerobic growth conditions. * *p <* 0.05 and *** *p <* 0.001 compared with vehicle (**a**–**e**) and MRSA (f). # *p <* 0.05 compare with each dose of temoporfin.

The biofilm removal assay revealed that the concentration of 2 μg/mL temoporfin had noticeably high biofilm removal efficacies against biofilms of *A. actinomycetemcomitans*, *E. faecalis*, and *S. aureus*. However, it had no apparent effect on biofilms of *L. acidophilus*, *L. paracasei*, *S. mutans*, and MRSA biofilms under normoxic conditions (Figure 5a). When the concentration of temoporfin was 8 μg/mL, there was only a slight removal effect on biofilms of *L. acidophilus*, *L. paracasei*, and MRSA.

#### 2.4.2. The Biofilm Removal Effect of Temoporfin and KI Co-Treatment in a Hypoxic Environment

Hypoxic conditions enhanced the resistance to most antibiotics in the pathogens [30]. In the hypoxic environment, at concentrations of 1, 2, 4, and 8 μg/mL, temoporfin combined with 1 mg/mL KI did not demonstrate substantial removal efficacy against preformed MRSA biofilms (Figure 5b). Notably, 1 μg/mL temoporfin combined with 1 mg/mL KI had high biofilm removal efficacies against *L. acidophilus* and *L. paracasei* biofilms in the hypoxic environment (Figure 5c,d). This confirmed that the synergistic activity of KI and temoporfin can enhance the biofilm removal effect compared with temoporfin alone in vitro.

#### 2.4.3. The Effect of Temoporfin on the Expression of Genes Regulating Biofilm Formation in MRSA

The transcription levels of genes (*agrA*, *icaA*, *sarA*, and *srtA*) associated with MRSA biofilms formation were determined by reverse transcription-quantitative real-time polymerase chain reaction (RT-qPCR) (Figure 5e). The expressions of *agrA*, *icaA,* and *sarA* were downregulated by 0.125 μg/mL temoporfin treatment. However, *srtA* was upregulated by 1 μg/mL temoporfin treatment. The expression of *sarA* was not affected by temoporfin treatment. However, the temoporfin treatment and temoporfin and KI co-treatment did not show the MRSA-biofilm removal activity in normoxia (Figure 5a) and hypoxia (Figure 5b), respectively.

#### 2.4.4. Endogenous Hydrogen Peroxide Production in the Species That Were Sensitive to Temoporfin and KI Co-Treatment

KI and H_2_O_2_ reactions produce oxygen. It was unclear whether temoporfin utilized KI-produced oxygen to enhance antibacterial activity. To clarify why the synergistic effect of temoporfin and KI was limited in *L. acidophilus* and *L. paracasei*, we tested the amounts of H_2_O_2_ produced by MRSA, *L. acidophilus*, and *L. paracasei* under hypoxic conditions (Figure 5f). The amount of H_2_O_2_ produced was 12.36 ± 0.7248, 29.34 ± 1.446, and 35.64 ± 3.888 μM for MRSA, *L. acidophilus*, and *L. paracasei*, respectively. *L. acidophilus* and *L. paracasei* produced more H_2_O_2_ than MRSA (*p* < 0.05), and this was consistent with the synergistic effect observed only in *L. acidophilus* and *L. paracasei.*

## 3. Discussion

The effect of PDT depends on the absorption of photosensitizers, the light energy, and the intracellular oxygen content. At temoporfin MIC, the bacteria absorbed the photosensitizer for 3 h, and the best effect was obtained after 15 min of illumination. Additionally, the effect was weakened before this time-point (Figure 1c). In addition, the experimental results confirmed that temoporfin irradiation could be conducted multiple times. When 0.5 μg/mL temoporfin was administered for 5 min, the bacteria were not inhibited in the experiment based on method II but were inhibited in the experiment based on method III (Figure 1d). Method III involved exposure for 5 min a day for three consecutive days, and more temoporfin accumulated in the bacteria to suppress bacterial growth. The antibacterial effect of temoporfin was more pronounced. However, the long waiting time for absorption and the long irradiation time may limit the clinical application of aPDT.

KI is a biocompatible compound that is readily available, safe, and effective as an inorganic salt. A high dosage of KI still reduced *S. mutans* (Figure 2d), *L. acidophilus*, and *L. paracasei* growth (Figure 3b) instead of *A. actinomycetemcomitans* (Figure 2c). In the study, *A. actinomycetemcomitans* is a Gram-negative bacterium, and the others are Gram-positive bacteria. The Gram-positive species have a thick and porous peptidoglycan cell wall surrounding a cytoplasmic membrane. The Gram-negative species have double lipid bilayers sandwiched between the peptidoglycan layer. Small molecules’ penetration into Gram-positive species are easier than Gram-negative species [31]. The impermeable external membrane of the Gram-negative bacteria cell wall limits the anionic or neutral-charge molecule entrance [32]. Therefore, we supposed that the stress resistance of *A. actinomycetemcomitans* to KI is higher than that of *S. mutans* due to diverse cell wall structures. The effect of KI on Gram-positive bacteria is more obvious than that on Gram-negative bacteria.

In this study, *A. actinomycetemcomitans* was the only Gram-negative bacterium but did not show the worst response to temoporfin. The Gram-positive strains in this study—*E. faecium* and *S. aureus*—showed high sensitivity to temoporfin, but MRSA did not (Table 1). The biofilm removal effect of temoporfin was weak under the normoxic environment (Figure 5a). As temoporfin does not completely inhibit the genes associated with the formation of *MRSA* biofilms, there was no significant effect observed for biofilm removal under hypoxic conditions (Figure 5b,e). Ampicillin and CHX were antibiotic and antiseptic, respectively. Both ampicillin and CHX were commonly used in clinical dentistry. They interfered with the synthesis of the cell wall by different mechanisms. A high dose of CHX can disrupt the cell membrane and cause cell death. However, long-term usage of ampicillin and CHX will induce several side effects, such as antibiotic resistance, rash, nausea, diarrhea, skin irritation, teeth discoloration, and allergic reactions. *Lactobacilli* have been associated with dental caries [33]. Temoporfin and ampicillin combination and temoporfin and CHX combination show the synergistic effect of antibacterial activity in MRSA (Figure 4). The temoporfin suppressed the expression of the drug resistance gene *mecA* and helped reduce the working dosage of ampicillin and CHX. Therefore, a low dose of temoporfin, ampicillin, CHX, and KI co-treatment can be used to reduce the *Lactobacilli* cell numbers and biofilms for caries prevention and therapy.

The present study results show that in normoxic and hypoxic environments, *A. actinomycetemcomitans*, *S. mutans*, *E. faecalis*, *S. aureus*, and MRSA can be completely inhibited by 1–8 μg/mL temoporfin but that *L. acidophilus* and *L. paracasei* cannot. However, the addition of KI enhanced the effect of temoporfin, especially against *Lactobacillus acidophilus* and *Lactobacillus paracasei* (Table 1, Figure 3c,d and Figure 5c,d). In normoxic and hypoxic environments, the MIC and MBC of *L. acidophilus* and *L. paracasei* were reduced by adding 1 mg/mL KI (Table 1). In synergistic analysis, we observed that the growth of *L. acidophilus* treated with 8 μg/mL temoporfin combined with 1 mg/mL KI significantly decreased. In addition, the growth of *L. paracasei* treated with 0.5 μg/mL temoporfin combined with 0.25 mg/mL KI significantly decreased (Figure 3). The reaction appears to be the addition of iodide and singlet oxygen to produce reactive peroxy iodide and hydrogen peroxide, producing a stable antimicrobial substance—iodine or tri-iodide. The chemical reaction is represented as follows [24,34]:

^1^O_2_ + I^−^ → IOO^−^

IOO^−^ + H^+^ → HOOI

IOOH + I^−^ → HOOI_2_^−^

HOOI_2_^−^ → I_2_^−^ + HOO

^1^O_2_ + 3I^−^ + 2H_2_O → I_3_^−^ + 2H_2_O_2_

These bactericidal components are probably responsible for the long-term bactericidal effect that persists after ceasing illumination [27]. Although *L. paracasei* and *L. acidophilus* were sensitive to the above active substances, KI did not distinctly enhance the photobactericidal effect of temoporfin on all test bacteria in our study. Thus, there are still other bacterial endogenous factors affecting the photobactericidal effect.

The Fenton reaction is a process of advanced oxidation during which the ferrous ion (Fe^2+^) is oxidized by hydrogen peroxide (H_2_O_2_) to the ferric ion (Fe^3+^), forming a hydroxyl radical (OH^•^) and a hydroxide ion (OH^−^) in the process [35]. Only *L. acidophilus* and *L. paracasei* were cultured in de Man, Rogosa, and Sharpe (MRS) broth. In contrast, the other bacteria were cultured in brain heart infusion (BHI) and tryptic soy broth (TSB). We reconfirmed the composition of the MRS broth and TSB. There was no iron (Fe) component in either broth; therefore, we excluded the Fenton reaction. It was thus confirmed that in TSB, the H_2_O_2_ produced by MRSA was not utilized by the Fenton reaction.

Under aerobic conditions, bacterial pyruvate metabolized carbon via pyruvate oxidase to form H_2_O_2_ [36]. H_2_O_2_ production decreased by two- to three-fold when certain bacteria were grown in a hypoxic environment. *Lactobacilli* produced bacteriocins, lactic acid, and H_2_O_2_ to suppress the pathogenic growth of certain bacteria [37]. Regarding H_2_O_2_ production in this study, we observed that *L. acidophilus* and *L. paracasei* produced more H_2_O_2_ than MRSA did under a hypoxic environment (Figure 5f). Illuminated photofrin in the presence of KI produced hydrogen peroxide but not superoxide [29]. This evidence supports that KI enhances oxygen generation to promote the photobactericidal activity of temoporfin through endogenous and chemical productions reaction of H_2_O_2_. However, further research is needed to determine the pyruvate oxidase activity of *L. acidophilus*, *L. paracasei*, and MRSA strains. In addition to KI, manganese peroxide (MnO_2_) and copper oxide (CuO) can also generate oxygen by reacting with H_2_O_2_. MnO_2_ or CuO reaction with H_2_O_2_ is relatively slower than KI, and it is unclear whether they can promote the effect of temoporfin. The effects of MnO_2_ and CuO on bacteria or cells need to be evaluated.

## 4. Materials and Methods

### 4.1. Microorganisms Culture

*A. actinomycetemcomitans* (ATCC 33384), *S. mutans* (ATCC 25175), *E. faecalis* (BCRC 10789), *L. acidophilus* (BCRC 10695), *L. paracasei* (BCRC 16093), *S. aureus* (ATCC 25923), and MRSA (ATCC 43300) were used in this study. *A. actinomycetemcomitans* was cultured in BHI broth. *S. mutans*, *E. faecalis*, *S. aureus*, and MRSA were cultured in TSB. *L. acidophilus* and *L. paracasei* were cultured in MRS broth. The bacteria were inoculated by loop transfer from frozen tubes into 3 mL of nutrient broth slant, and they were incubated at 37 °C for 24 h with constant shaking at 200 rpm. Bacteria from these cultures were transferred to the appropriate agar plates and incubated overnight. The selected single colony was transferred to a suitable liquid medium and incubated for 4–6 h to achieve logarithmic growth. The OD600 of each culture was adjusted to 1.0 using fresh broth to achieve a standard inoculum of 10^6^ CFU/mL. Stock cultures were maintained at –80 °C in a growing broth containing 25% sterile glycerol [38,39].

### 4.2. Determination of Temoporfin Conditions, MIC, and MBC

Cell suspensions were prepared by inoculating 2 mL of 10^6^ CFU/mL microbes from each logarithmic phase stock into 2 mL of broth containing various concentrations of the test compounds in 15 mL culture tubes. Temoporfin (ChemScene, Monmouth Junction, NJ, USA) was dissolved in dimethyl sulfoxide (DMSO) as 100 mg/mL stock solutions and stored at −20 °C. The bacterial suspensions were treated with various doses of temoporfin and incubated at 37 °C for 24 h with constant shaking at 200 rpm. Temoporfin treatments were used in conjunction with a diode laser (TI-818-1, Transverse Industries Co., Ltd., New Taipei City, Taiwan), a red light source with emission at 635 ± 5 nm. The device is designed with four independent light sources for laboratory use only [40,41]. The distance from the light to the sample was 15 cm, and the spot size diameter was 5.5 cm. The exposure times were 3, 5, 10, and 15 min (2–10 J/cm^2^) or daily exposure for 5 min for 3 days. The concentration at which no visible turbidity was observed represented the MIC. It was subsequently inoculated on sterile 10 cm nutrient agar plates with no test compound and incubated for 24 h. The lowest concentration of the test compound with no growth was considered the MBC [38].

### 4.3. Growth Curve Assay

Growth curve analysis was performed in a 96-well format adapted from a previously described method [42]. Bacterial suspensions were prepared by inoculating 1 μL of 10^6^ CFU/mL microbes from each logarithmic phase stock in 1 mL of the liquid medium containing various concentrations of temoporfin and potassium iodide (KI) (Sigma-Aldrich^®^, St. Louis, MO, USA) in 15 mL culture tubes. After 3 h of incubation, the bacterial suspension was transferred to a 3 cm culture dish and exposed to 635 nm red light for 15 min (2–10 J/cm^2^). The bacterial suspension (200 μL) was transferred to 96-well plates for testing, and 200 μL of sterile liquid broth was used as a blank. The 24-h growth curve analyses were performed for *A. actinomycetemcomitans* and *S. mutans* at 37 °C. The kinetic analysis included a 5 s shaking step before each of the OD600 time point measurements, which were recorded at 30 min intervals. The concentration was analyzed using a VersaMax™ ELISA microplate reader (Molecular Device, San Jose, CA, USA) and Softmax^®^ Pro (version 5.4.1) software.

### 4.4. Synergistic Effect Test

Log phase of *L. acidophilus* and *L. paracasei* suspensions were prepared in 2 mL of TSB in 15 mL culture tubes by inoculating 2 μL of 10^6^ CFU/mL microbe from each logarithmic phase stock and incubated at 37 °C for 4–6 h at 200 rpm. The OD600 was adjusted to 1.0 using fresh broth to obtain *L. acidophilus* and *L. paracasei* suspensions of 10^6^ CFU/mL. *L. acidophilus* and *L. paracasei* suspensions were treated with temoporfin (0.5, 1, 2, 4, and 8 μg/mL) or KI (0.25, 0.5, 1, 2, and 4 mg/mL), or co-treated with temoporfin (0.5, 1, 2, 4, and 8 μg/mL) and KI (0.25, 0.5, and 1 mg/mL) for 24 h for the synergistic test. The cultures were incubated at 37 °C for 24 h at 200 rpm, and OD600 was measured for ten-fold dilutions of each culture [43].

### 4.5. Reverse Transcription (RT)-qPCR

Bacteria (10^6^ CFU/mL) were inoculated into medium containing temoporfin concentrations of 0.125, 0.25, 0.5, and 1 μg/mL and incubated for 24 h. The microbes were collected for reverse transcription-polymerase chain reaction (RT-PCR) analysis. The total ribonucleic acid (RNA) of cells treated with the drug was extracted using TRI Reagent (Molecular Research Center, Inc., Cincinnati, OH, USA). RT of total RNA was conducted using a random primer, and complementary deoxyribonucleic acid (cDNA) was used as the PCR template. The expression of antibiotic resistance-related genes (*mecI*, *mecR1*, and *mecA*) and biofilm formation-related genes (*agrA*, *icaA*, *sarA*, and *srtA*) was normalized to glyceraldehyde 3-phosphate dehydrogenase (*GAPDH*) expression [38].

### 4.6. Biofilm Removal Assay

Bacteria (10^6^ CFU/mL) were inoculated in a 96-well plate and incubated at 37 °C for 24 h to produce mature bacterial biofilms. After removing the planktonic bacteria, the biofilms were washed once with sterile phosphate-buffered saline (PBS). Then 100 μL of medium containing various concentrations of temoporfin and KI was added to each well. All treatment groups were exposed to a diode laser for 15 min. After 4 h of incubation at 37 °C, the medium was aspirated, and then the wells were washed with PBS twice and air-dried for 1 h. Crystal violet (150 μL of 0.1% *w*/*v*) was added to each well and left to stand at room temperature for 10–15 min. The crystal violet was aspirated, and the plate was rinsed four times with water. After aspirating water, 150 μL of 33% acetic acid was added to each well. Absorbance was determined at 550 nm on the VersaMax™ ELISA microplate reader using 30% acetic acid in water as the blank [40,44,45].

### 4.7. Hydrogen Peroxide Production Assay

Endogenous peroxide production was analyzed by a spectrophotometric assay, with reference to the experiment of Pericon et al. [36]. Under anaerobic conditions, selected colonies were transferred to a suitable liquid medium and incubated for 4–6 h to achieve logarithmic growth. The OD600 was adjusted to 0.6 using fresh broth to obtain bacterial suspensions, which were incubated for 2 h; then, the OD600 was adjusted to 1.0 using fresh broth. Cells were centrifuged at 4 °C for 20 min at 4000× *g*, washed twice in ice-cold PBS (pH = 7.4), and resuspended in PBS with 0.5 mM glucose to attain twice the original culture volume. H_2_O_2_ production was measured in PBS to minimize the Fenton reaction. After 1 h of incubation at 37 °C under anaerobic conditions, the cultures were collected by centrifugation for 10 min at 10,000× *g* and filtered through a 0.2 μm (pore size) membrane. Before measuring H_2_O_2_ production, phenol red (Ishizu Pharmaceutical Co., Ltd., Osaka, Japan) and horseradish peroxidase (Sigma-Aldrich^®^, St. Louis, MO, USA) were added to the peroxide assay buffer at final concentrations of 0.46 mM and 0.046 U/mL, respectively. An aliquot of the filtered supernatant was added to the assay mixture at a ratio of 1 to 4 and incubated for 30 min at 37 °C. NaOH (final concentration of 0.004 N) was added to stop the reaction, and the absorbance was recorded at 610 nm. Concentrations were computed using a standard curve with known amounts of H_2_O_2_.

### 4.8. Statistical Analysis

Statistical data were obtained from three independent experiments. Data are shown as the mean ± standard error. Statistically, significant differences were determined using one-way ANOVA and paired *t*-test using Prism 5.0 (GraphPad Software, Inc., La Jolla, CA, USA). Differences between variants were considered significant at *p* < 0.05. CompuSyn software (Version 1.0, ComboSyn Inc., Paramus, NJ, USA) was used to quantify the synergism and antagonism of the drug combinations.

## 5. Conclusions

The current study investigated the MIC and MBC values of temoporfin against common oral microbes under normoxic and hypoxic conditions. The antibacterial activity of temoporfin was more effective under normoxic conditions than under hypoxic conditions. The combination of temoporfin with KI had synergistic effects of suppressing bacterial growth and enhancing biofilm removal activity in *L. acidophilus* and *L. paracasei.* They produce more H_2_O_2_ than MRSA under hypoxic conditions. The combination of temoporfin with ampicillin or CHX also showed synergistic effects for reducing the antibiotic-resistant ability of MRSA. Since PDT treatment is expensive, reducing PDT dose and improving photosensitivity can help reduce medical costs. The combination of temoporfin and KI, as well as temoporfin and antibacterial agents, could be an effective remedy for treating oral and systemic diseases.

## Figures and Tables

**Table 1:** The MIC/MBC of the oral pathogens after temoporfin and the combination of 1 mg/mL KI treatment under normoxic and hypoxic conditions.

	Normoxia (MIC/MBC) (μg/mL)		Hypoxia (MIC/MBC) (μg/mL)	
Oral Bacteria	Temoporfin	Temoporfin + 1 mg/mL KI	Temoporfin	Temoporfin + 1 mg/mL KI
*A. actinomycetemcomitans* (G−)	4/4	4/4	4/8	2/4
*E. faecalis* (G+)	1/1	1/1	1/1	1/2
*L. acidophilus* (G+)	>8/>8	2/4	>8/>8	2/4
*L. paracasei* (G+)	>8/>8	2/3	>8/>8	2/3
*S. aureus* (G+)	1/2	1/1	2/2	1/1
MRSA (G+)	8/8	4/8	8/8	4/8
*S. mutans* (G+)	2/2	0.5/1	2/2	2/2

## Data Availability

The data presented in this study are available in article.
